# The MAPKKK CgMck1 Is Required for Cell Wall Integrity, Appressorium Development, and Pathogenicity in *Colletotrichum gloeosporioides*

**DOI:** 10.3390/genes9110543

**Published:** 2018-11-08

**Authors:** Yu-Lan Fang, Li-Ming Xia, Ping Wang, Li-Hua Zhu, Jian-Ren Ye, Lin Huang

**Affiliations:** Co-Innovation Center for Sustainable Forestry in Southern China, Nanjing Forestry University, Nanjing 210037, Jiangsu, China; fangyulan19921028@gmail.com (Y.-L.F.); summerxlm@163.com (L.-M.X.); pingwangnjfu@gmail.com (P.W.); lhzhu@njfu.edu.cn (L.-H.Z.); jrye@njfu.edu.cn (J.-R.Y.)

**Keywords:** mitogen-activated protein kinase cascades, cell wall integrity, appressorium, pathogenicity

## Abstract

Mitogen-activated protein kinase (MAPK) signaling pathway plays key roles in sensing extracellular signals and transmitting them from the cell membrane to the nucleus in response to various environmental stimuli. A MAPKKK protein CgMck1 in *Colletotrichum gloeosporioides* was characterized. Phenotypic analyses of the ∆*Cgmck1* mutant showed that the CgMck1 was required for vegetative growth, fruiting body development, and sporulation. Additionally, the *CgMCK1* deletion mutant showed significant defects in cell wall integrity, and responses to osmotic stresses. The mutant abolished the ability to develop appressorium, and lost pathogenicity to host plants. The ∆*Cgmck1* mutant also exhibited a higher sensitivity to antifungal bacterium agent *Bacillus velezensis*. The deletion mutants of downstream MAPK cascades components CgMkk1 and CgMps1 showed similar defects to the ∆*Cgmck1* mutant. In conclusion, CgMck1 is involved in the regulation of vegetative growth, asexual development, cell wall integrity, stresses resistance, and infection morphogenesis in *C. gloeosporioides*.

## 1. Introduction

Fungal mitogen-activated protein kinase (MAPK) cascades play key roles in determining fungal development and responses to a variety of stress stimuli [1]. Several distinct MAPK kinase cascades exist in *Saccharomyces cerevisiae*, which regulate mating (Fus3/Kss1), response to high osmotic stresses (Hog1) and maintenance of cell wall integrity (CWI) (Slt2) [2]. The CWI pathway remains as one of the key pathways controlling the cellular remodeling process in response to internal cues and environmental challenges [3,4]. The core of this signal transduction pathway is a MAPK cascade consisting of MAPKKK protein Bck1/Mck1, MAPKK protein Mkk1/2 and MAPK protein Slt2/Mps1. This pathway is activated through the activation of Rho1 and the phosphorylation of Pkc1 for sequential phosphorylation of Bck1, Mkk1/2 and Slt2 [5,6].

Previous studies showed that CWI pathway genes are involved to regulate invasive structures development and virulence to plant hosts in phytopathogenic fungi. For example, *Magnaporthe oryzae* MoMck1 is required for maintenance of CWI, conidiogenesis, and pathogenicity [7]. Disruption of *MoMKK1* results in less aerial hyphae, defective asexual development and attenuated pathogenicity. In addition, MoMkk1 is involved in the osmotic stress response and the maintenance of CWI [8]. Mps1 plays an important role in CWI, stress response and pathogenicity in *M. oryzae* [9]. In *Fusarium oxysporum*, deletion of FoBck1, FoMKK2 and FoSlt2, respectively, resulted in attenuated pathogenicity and slower growth rate [10]. MAPK AaSlt2 regulates sporulation, melanin production and virulence in *Alternaria alternata* [11]. These findings suggested significant roles of CWI MAPK signaling pathway in multiple physiological processes in different microorganisms. Inhibition of the CWI MAPK signaling pathway will disturb infection progresses and facilitate the efficient control of phytopathogenic fungi.

*Colletotrichum gloeosporioides* is a ubiquitous plant pathogen infecting a wide range of plant species and causes enormous economic losses worldwide [12,13]. *C. gloeosporioides* infects host plants by a specialized infection structure called appressorium [14]. MAP kinase cascades have been confirmed to involve in the appressorium formation and virulence. CgPKA is required for appressorium formation and virulence [15]. The *CgMEK1*/*CgMKK1* deletion mutants showed the defects in appressorium formation and pathogenicity [16]. Previous study also explored that CgSlt2/CgMps1 play important roles in maintenance of CWI and regulating virulence to host plants in *C. gloeosporioides* [17]. Despite the function of CWI MAP kinase kinase CgMEK1/CgMkk1 and MAP kinase CgSlt2/CgMps1 has been reported, the upstream component of CWI MAPK pathway protein CgMck1 in *C. gloeosporioides* is still not known yet. 

In this study, we identified a *S. cerevisiae* Bck1 homologue, CgMck1, in *C. gloeosporioides*. CgMck1 is not only required for vegetative growth, cell wall integrity and osmotic stress response, but also involved in regulating sporulation, appressorium development and pathogenicity in *C. gloeosporioides*.

## 2. Materials and Methods

### 2.1. Strains and Culture Conditions

*Colletotrichum gloeosporioides* sensu stricto (s.s.) SMCG1#C isolated from the diseased leaves of Chinese fir with symptoms of anthracnose [18] and supplied by Forest Pathology Lab of Nanjing Forestry University (Nanjing, China) was used as the wild type strain. The wild type, gene deletion mutants and the complemented strains used in this study were maintained on the potato dextrose agar (PDA) medium plates at 25 °C. Liquid complete medium (CM) medium was used to culture fungal mycelia for genomic DNA extraction, and protoplasts preparation [19].

### 2.2. Targeted Gene Deletion and Complementation

Based on the genome draft sequence of *C. gloeosporioides* s.s. SMCG1#C [20], the *CgMCK1* gene replacement constructs were established using the overlap polymerase chain reaction (PCR) method as described [13]. Firstly, the upstream (~1.5 kb) and downstream (~1.5 kb) flanking sequences were amplified with primer set CgMCK1_F1/R1 and CgMCK1_F2/R2, respectively. The fragments of hygromycin phosphotrasferase (*HPH*) cassette were amplified with primer set HYG_F/R. Secondly, the upper and downstream flanking sequences were fused to *HPH* cassette with a primer set CgMCK1_F1/HY_R and YG_F/CgMCK1_R2 using overlap PCR, respectively. Thirdly, a 3.8-kb gene replacement fragment were amplified with a primer set CgMCK1_F3/R3 and purified and transformed into the protoplasts of wild type *C. gloeosporioides* SMCG1#C as described [18]. The deletion mutant of *CgMCK1* was confirmed by Southern blot analysis using a method previously described [21] with the hybridization probes of *CgMCK1* and *HPH*, respectively.

For complementation, an 8.3-kb fragment containing the *CgMCK1* gene coding region and its native promoter region were amplified from the wild type genomic DNA using a primer set CgMCK1_ComF/R. The resulting PCR products were purified and co-transformed into the mutant protoplasts with pYF11 vector. The transformants were selected on TB3 (0.3% yeast extract, 0.3% casamino acids, and 20% glucose) agar medium amended with 400 mg mL^−1^ ppm geneticin (Gibco, Life Technologies, Carlsbad, CA, USA) and checked by PCR amplification using a primer set CgMCK1_InnerF/R (Supplementary Materials Table S1). PCRs were performed in an Eppendorf Nexus Thermal Cycle (Eppendorf, Hamburg, Germany). *CgMKK1* and *CgMPS1* gene deletion mutants and their complemented strains were obtained using a similar strategy like *CgMCK1*. Primers were designed using the Primer Premier 5.0 software (Premier Biosoft International, Corina Way, CA, USA), and were synthesized by GenScript Biotech Corp. (Nanjing, China). The primer sequences used in this study were listed in the Table S1.

### 2.3. Vegetative Growth and Fruiting Body Development Assays

Mycelial plugs of the wild type, the gene deletion mutants and the complemented strains were inoculated onto CM, PDA, Mathur’s agar, and minimal medium (MM), respectively. Plates were kept in an incubator at 25 °C and colony diameter was measured at 5 days post inoculation [22]. Fruiting bodies were induced to develop on V8 medium plates [19] after inoculation for 10 days at 25 °C under 16 h/8 h light and dark cycle.

### 2.4. Various Stresses Resistance and Protoplast Release Assays

Mycelial plugs were inoculated onto the CM agar plates with sodium dodecylsulfate (SDS) (0.005%), Calcofluor white (CFW) (50 and 100 µg mL^−1^), Congo red (CR) (200 and 600 μg mL^−1^), NaCl (0.7 M), sorbitol (1 M) and cultured in the dark for 5 days at 25 °C. Hyphal growth inhibition rate was calculated using the method described previously [22].

For protoplast release assay, hyphae of wild type, mutant and complemented strains were cultured in liquid CM, respectively, and shaken at 118 rpm for 48 h at 25 °C. The hyphae were collected using two layers of Miracloth (EMD Millipore, Billerica MA, USA). Then the excessive water was removed with filter paper. A hundred mg of hyphae were used for monitoring protoplast release as described previously [18].

### 2.5. Sporulation, Conidial Germination and Appressorium Formation

For sporulation, the plugs of fungal strains were inoculated in liquid CMC medium [18] and the culture was shaken at 150 rpm for 48 h at 25 °C followed by filtering through two layers of Miracloth. Filtrate was centrifuged at 7000 rpm for 8 min using an Eppendorf 5804R centrifuge (Eppendorf, Hamburg, Germany), and the centrifugate was washed three times with distilled water after supernatant was decanted.

For conidial germination and appressorium formation, 20 µL of conidial suspension at a concentration of 10^5^ conidia mL^−1^ were placed on hydrophobic cover slips and incubated at 25 °C as previously described [18]. To observe the penetration and invasive hyphae, 10 µL of the conidial suspension were inoculated onto the onion epidermal layers and observed at 18 h post inoculation. At least 30 measures per structure were measured under a ZEISS Axio Imager A2m microscope (Carl Zeiss, Göttingen, Germany).

### 2.6. Plant Infection Assays

Conidia of the wild type, the mutants and complemented strains were prepared in CMC medium as afore-described. Conidial suspension was adjusted to 1 × 10^5^ spore mL^−1^. Ten μL of conidial suspension were inoculated onto the detached leaves of Chinese fir (*Cunninghamia lanceolata*) and poplar (*Polulus* × *euramericana* cv. Nanlin 895), respectively. The inoculated leaves were kept under moist condition and incubated in a chamber at 25 °C under a 12-h light/dark cycle [14]. Lesions on leaves of Chinese fir and poplar were observed at four days and five days post inoculation, respectively.

### 2.7. Sensitivity Examination of Gene Deletion Mutants against Biocontrol Agents

In order to examine the sensitivity of the wild type, the targeted gene deletion mutants and complemented strain against biocontrol agents, a mycelial plug of the wild type, the mutants and the complemented strains were placed in the center of a 9-cm PDA plates, respectively. The biocontrol agents including *Bacillus velezensis* isolate #22 and *Epicoccum* sp. isolate ENML1 were inoculated at the sites three cm away from the pathogen disc in two perpendicular directions in the same plate [23]. Plates were incubated at 25 °C until inhibition zones were observed. Hyphal growth inhibition was quantified by measuring the radius of the pathogen colony in the direction of the antagonist colony, and calculated as the percentage of inhibition of radial growth according to the method reported before [23].

### 2.8. Microscopic Observation and Statistical Analysis

Lesions and invasive hyphae on the detached leaves of poplar were stained using Tryphan blue according to the method as described previously [24]. Photographs were taken under a Zeiss M2 microscope (Leica Microsystems, Wetzlar, Germany). All experiments were carried out at least three times, and each treatment had three replicates. Statistical analyses were performed with the SPSS 19.0 software program (SPSS Inc., Chicago, IL, USA) using a one-way analysis of variance (ANOVA) followed by LSD’s multiple range tests.

## 3. Results

### 3.1. Deletion of *CgMCK1* and Reintroducing *CgMCK1* into *∆Cgmck1* Mutant

**Figure 1:**
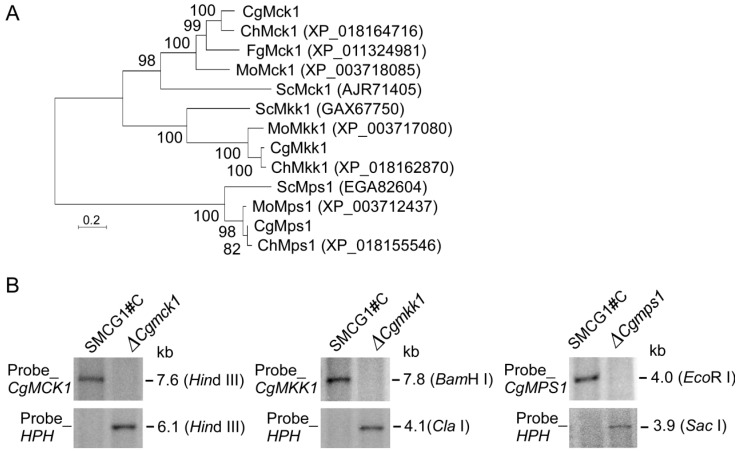
Phylogenetic tree of fungal MCK1 and southern blot analysis of the targeted gene deletion mutants. (**A**) Phylogenetic tree generated using the Mega7.0 program with neighbor-joining method (https://www.megasoftware.net/). ChMck1, ChMkk1 and ChMps1 form *Colletotrichum higginsianum*. MoMck1, MoMkk1 and MoMps1 from *Magnaporthe oryzae*. ScMck1, ScMkk1 and ScMps1 from *Saccharomyces cerevisiae*. FgMck1 from *Fusarium graminearum*. (**B**) Southern blot analysis of the targeted gene deletion mutants.

We identified a homolog in *C. gloeosporioides* genome database and found that 61% amino acid sequence was shared with FgMCK1 in *Fusarium graminearum* (XP_011324981). The designated *CgMCK1* gene encodes a protein kinase of 1760 amino acids in length contained a putative protein kinase domain similar to the Mck1 of other fungi. The CgMCK1 was 51, 45, and 41% identical to the corresponding kinase domains of the fungal MCKs MoMCK1 from *M. oryzae*, UmMCK1 from *Ustilago maydis*, and Mck1/Bck1 from *S. cerevisiae*, respectively. Phylogenetic tree also demonstrated that CgMCK1 was the most similar to the MCK1 in microfungi and diverges from those of unicellular yeasts (Figure 1).

To investigate the roles of CgMck1 in *C. gloeosporioides*, a gene deletion mutant was generated by replacing the *CgMCK1* coding region with the hygromycin resistance (*HPH*) gene cassette. The mutant was confirmed by Southern blot analysis (Figure 1). For mutant complementation, the wild type *CgMCK1* gene with the native promoter was re-introduced into the ∆*Cgmck1* mutant and generated the complemented transformant ∆*Cgmck1*/*CgMCK1.* To explore whether the deletion of the *CgMCK1* gene is consistent with the phenotypes of the downstream *CgMKK1* and *CgMPS1* gene deletions in the MAPK signaling pathway, the ∆*Cgmkk1* and ∆*Cgmps1* mutants were obtained using a similar strategy (Figure 1).

### 3.2. CgMck1 Is Important for Vegetative Growth and Fruiting Body Development

**Figure 2:**
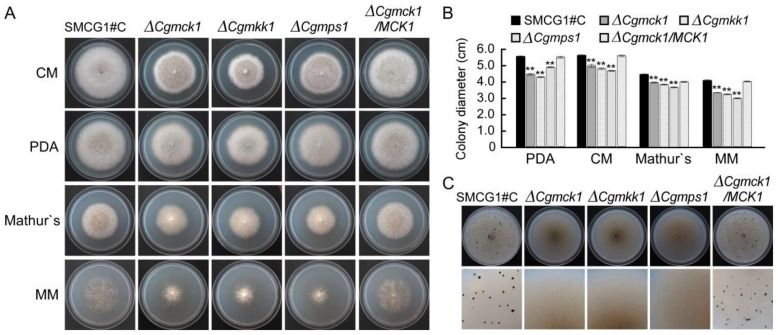
CgMck1 required for mycelia growth and fruiting body development. (**A**) Colony morphology of the wild type SMCG1#C, ∆*Cgmck1*, ∆*Cgmkk1*, ∆*Cgmps1* and the complemented strain ∆*Cgmck1*/*MCK1* grown on complete medium (CM), potato dextrose agar (PDA), Mathur’s and minimal medium (MM) media for five days. (**B**) Colony diameter of SMCG1#C, ∆*Cgmck1*, ∆*Cgmkk1*, ∆*Cgmps1* and the complemented strain ∆*Cgmck1*/*MCK1* grown on CM, PDA, Mathur’s and MM media for five days. Error bars represent the standard deviation (SD) and asterisks indicate statistically significant differences (*p* < 0.01). (**C**) Fruiting bodies formed on the V8 medium for ten days with 16 h light/8 h dark cycle.

To investigate the role of CgMCK1 in vegetative growth, the wild type SMCG1#C, the ∆*Cgmck1* mutant and the complemented strain ∆*Cgmck1*/*CgMCK1* were inoculated onto CM, PDA, Mathur’s and MM plates, respectively. Compared with the wild type and complemented strain, the ∆*Cgmck1* mutant showed a significant reduced colony diameter on various media, which was similar to the phenotypes of ∆*Cgmkk1* and ∆*Cgmps1* (Figure 2A,B). There was no any fruiting body formed on the V8 plates, which was similar to the ∆*Cgmkk1* and ∆*Cgmps1* mutants (Figure 2C). In contrast, a large number of fruiting bodies were observed on the plates inoculated by the wild type and complemented strain (Figure 2C). These results indicated that CgMck1 played crucial roles in regulating vegetative growth and fruiting body development in *C. gloeosporioides.*

### 3.3. CgMck1 Is Required for Cell Wall Integrity and Osmotic Stress Resistance

**Figure 3:**
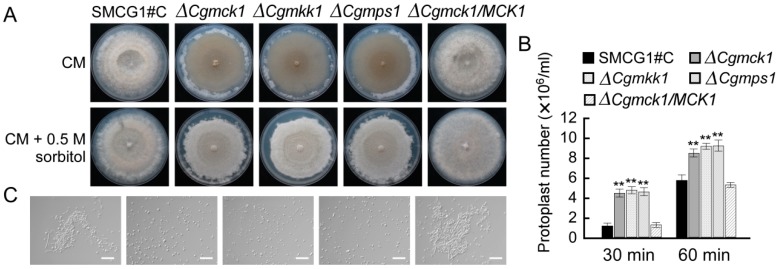
The ∆*Cgmck1* mutants showing defects in cell wall integrity. (**A**) Hyphal autolysis of the ∆*Cgmck1*, ∆*Cgmkk1*, ∆*Cgmps1* mutants observed on CM medium for 14 days post inoculation. (**B**) Protoplasts concentration at 30 min and 60 min after the mycelia was treated by wall cell-degrading enzyme. Error bars represent the SD, and asterisks indicate statistically significant differences (*p* < 0.01). (**C**) Protoplast production by cell wall degrading enzyme. Photographs were taken at 60 min post inoculation. Bars = 50 μm.

The cell wall sensitivity of the ∆*Cgmck1* mutant was tested on CM plates containing a variety of cell wall-perturbing agents including SDS, CFW, and CR. Compared to the wild type and complemented strains, the inhibition rate of the ∆*Cgmck1* mutant was significantly increased when exposed to SDS, CFW and CR. The similar results were also obtained in the ∆*Cgmkk1* and ∆*Cgmps1* mutants (Table 1). We also found that the ∆*Cgmck1* mutant exhibited the same hyphal autolysis to the ∆*Cgmkk1* and ∆*Cgmps1* mutants for 14 days post inoculation onto PDA plates. However, hyphal autolysis had not been observed in the wild type and complemented strain (Figure 3A). Addition of 0.5 M sorbitol restored the hyphal autolysis defect in the ∆*Cgmck1* mutant like the *MoMCK1* deletion mutant in *M. oryzae* [8]. These results suggested that CgMck1 may be involved in regulating the cell wall integrity. In order to test this hypothesis, we further investigated the effects of cell wall-degrading enzymes on hyphae of the wild type, the ∆*Cgmck1* mutant and the complemented strain. When hyphae were enzymatically cleaved, the ∆*Cgmck1* mutant released the protoplasts faster than the wild type and complemented strains as showed in Figure 3B,C.

We also tested the osmotic resistance of the ∆*Cgmck1* mutant and the results showed that the ∆*Cgmck1* mutant had lower growth inhibition than the wild type and the complemented strains on the medium plates containing 0.7 M NaCl. In the medium supplemented with 1 M sorbitol, the growth inhibition rate of ∆*Cgmck1* mutant was significantly higher than that of wild type and complemented strains (Table 1). These results collectively indicated that CgMck1 was required for maintenance of cell wall integrity and osmotic tresses resistance. The ∆*Cgmkk1* and ∆*Cgmps1* mutants also showed consistent results.

### 3.4. CgMck1 Is Essential for Sporulation and Appressorium Formation

**Figure 4:**
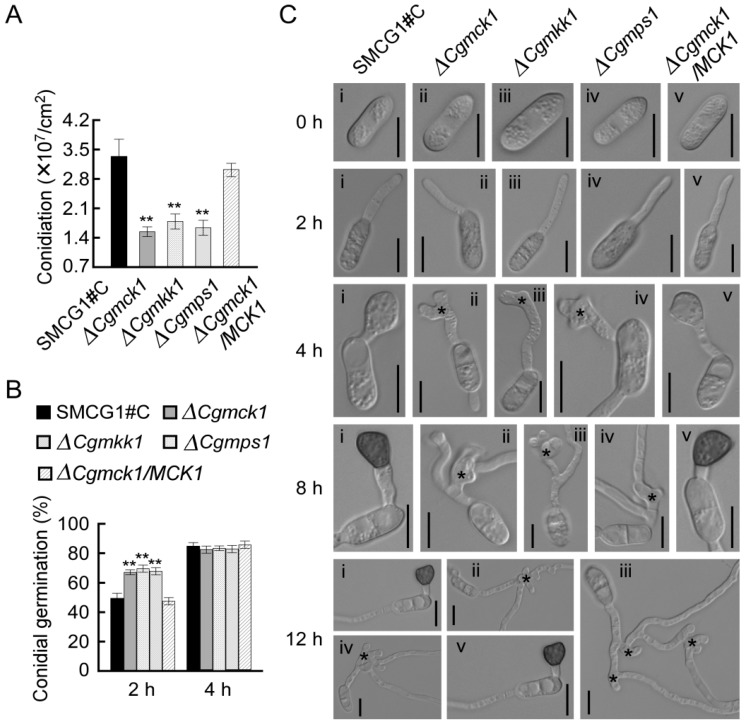
CgMck1 involving in sporulation, conidial germination, and appressorium development. (**A**) Sporulation of the wild type SMCG1#C, ∆*Cgmck1*, ∆*Cgmkk1*, ∆*Cgmps1*, and the complemented strain ∆*Cgmck1*/*MCK1* cultured in CMC medium for 48 h. Error bars represent the SD, and asterisks indicate statistically significant differences (*p* < 0.01). (**B**) Conidial germination rate of the wild type SMCG1#C, ∆*Cgmck1*, ∆*Cgmkk1*, ∆*Cgmps1*, and the complemented strain calculated after inoculation onto glass slides for 2 h and 4 h, respectively. Error bars represent the SD form the means, and asterisks indicate statistically significant differences (*p* < 0.01). (**C**) Appressorium development of the wild type SMCG1#C (i), ∆*Cgmck1* (ii), ∆*Cgmkk1* (iii), ∆*Cgmps1* (iv), and the complemented strain (v) at different hours post inoculation. Photographs were taken at 0, 2, 4, 8, 12 h after inoculation in vitro. Stars indicate the swollen cells. Bars = 10 μm.

Conidia play an important role for the infection of *C. gloeosporioies*. The wild type, the ∆*Cgmck1* mutant and the complemented strain were inoculated into CM medium to induce sporulation, respectively. Results showed that the ability for sporulation was significantly decreased in the ∆*Cgmck1* mutant than those of the wild type and the complemented strains (Figure 4A). We further evaluated the conidial germination rate in all strain. At 2 h, the ∆*Cgmck1* mutant had a higher germination rate than those of the wild type and the complemented strain. At 4 h, there was no significantly difference among these strains (Figure 4B). The data suggested that CgMck1 negatively regulated the conidial germination in the early stage in *C. gloeosporioides*. These results were also verified in both of the ∆*Cgmkk1* and ∆*Cgmps1* mutants.

*Colletotrichum gloeosporioides* infects plant cells depending on a specifically differentiated appressorium. In order to evaluate the effect of deletion of *CgMCK1* on appressorium formation, conidial suspension of these strains was placed onto the hydrophobic surface. At four hpi, a high proportion of swollen or hooked cells was formed at the tips of germtubes in the ∆*Cgmck1* mutant. Eight hours later, the swollen cell developed into vegetative hyphae-like structures (Figure 4C). By contrast, both of the wild type and the complemented strain produced typically-shaped appressoria at the end of germtubes at four hpi and melanized appressoria were formed at eight hpi. Until 12 hpi, there were no appressoria germinated in the wild type and the complemented strains (Figure 4C). These results indicated that CgMck1 is required for sporulation and appressorium development in *C. gloeosporioides*.

### 3.5. CgMck1 Is Required for Virulence of *Colletotrichum gloeosporioides*

**Figure 5:**
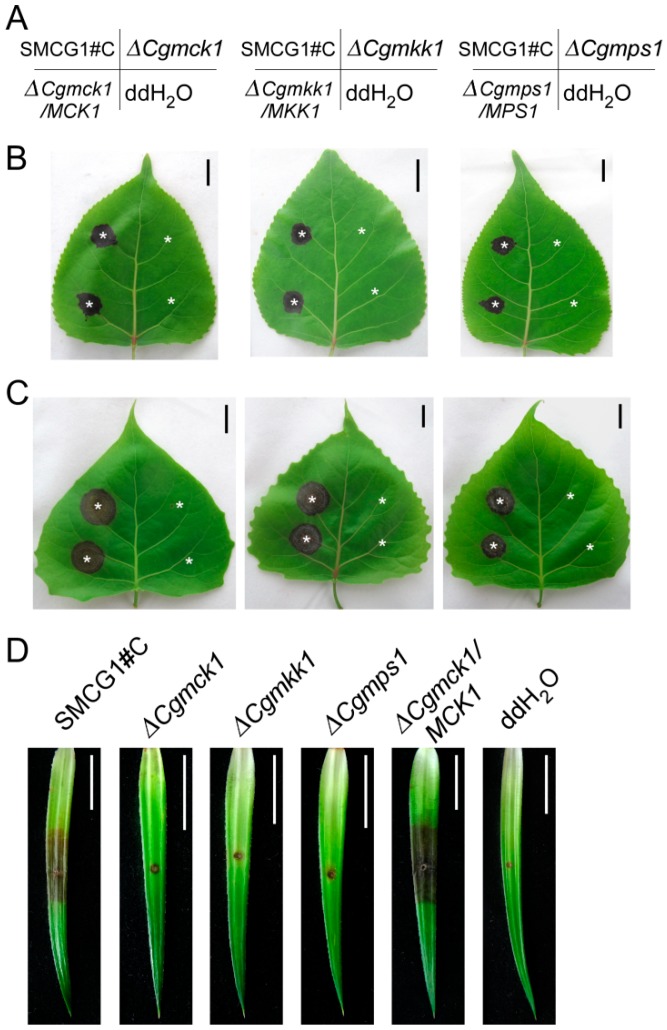
CgMck1 required for pathogenicity to host plant. (**A**) The conidial suspension inoculation position of the wild type SMCG1#C, ∆*Cgmck1*, ∆*Cgmkk1*, ∆*Cgmps1* and the complemented strains in picture B and C. (**B**) Pathogenicity assay on the unwounded detached leaves of poplar inoculated by the wild type SMCG1#C, ∆*Cgmck1*, ∆*Cgmkk1*, ∆*Cgmps1*, and complemented strain. (**C**) Pathogenicity assay on the wounded detached leaves of poplar inoculated by the wild type SMCG1#C, ∆*Cgmck1*, ∆*Cgmkk1*, ∆*Cgmps1*, and the complemented strains. Stars indicate the inoculation sites. (**D**) Pathogenicity assay on the wounded detached leaves of Chinese fir inoculated by the wild type SMCG1#C, ∆*Cgmck1*, ∆*Cgmkk1*, ∆*Cgmps1*, and complemented strains. Photographs were taken five days (**B**,**C**) and four days (**D**) after inoculation.

To determine whether the *CgMCK1* deletion mutant is pathogenic on the natural host, an equal number of conidia from the wild type, the ∆*Cgmck1* and the complemented strain were inoculated on the intact leaves of poplar. At 5 days post inoculation (dpi), typical lesions were observed on the unwounded leaves of poplar leaves inoculated by the wild type and the complemented strain, respectively. However, the ∆*Cgmck1* mutant caused almost no necrotic lesion on the leaves of poplar (Figure 5A,B). Under the similar condition, there was almost no lesion on the unwounded poplar leaves when inoculated by the ∆*Cgmkk1* and ∆*Cgmps1* mutants (Figure 5A,B).

In order to further determine whether the ∆*Cgmck1* mutant can infect the plant through wounds, an equal number of conidia were inoculated onto the wounded leaves of poplar and Chinese fir. The clear infection symptoms were observed on the leaves inoculated by the wild type and the complemented strain at 5 dpi, whereas no obvious necrotic lesion was found on the leaves inoculated by the ∆*Cgmck1* mutant (Figure 5A,C,D). The ∆*Cgmkk1* and the ∆*Cgmps1* mutants also did not show virulence to the host plants (Figure 5A,C,D).

**Figure 6:**
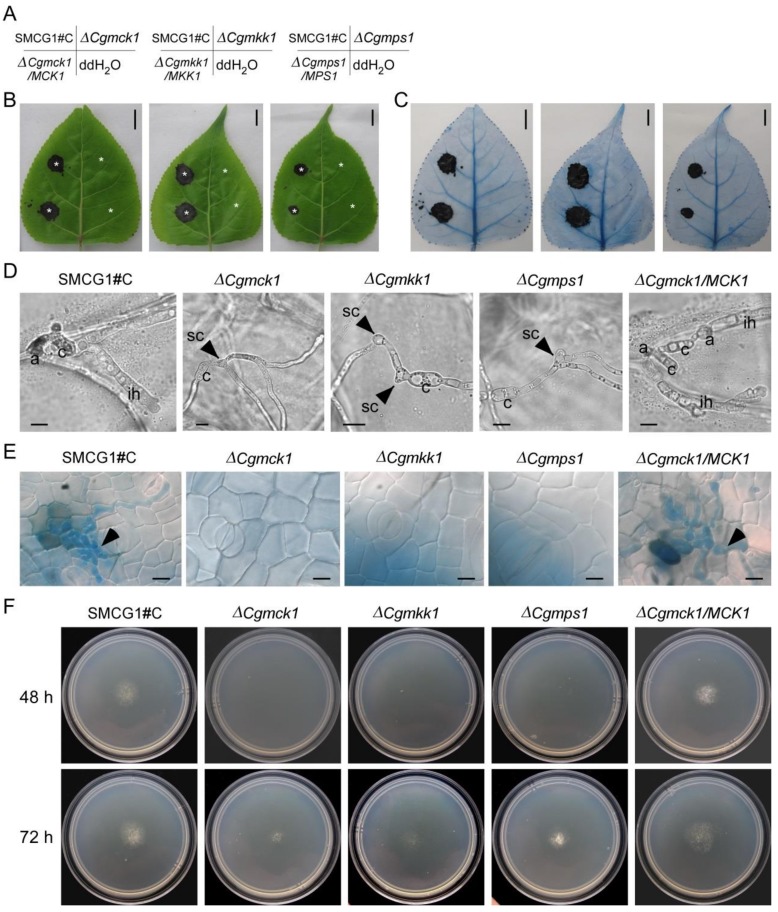
CgMck1 involving in penetration structures development. (**A**) The conidial suspension inoculation position of the wild type SMCG1#C, ∆*Cgmck1*, ∆*Cgmkk1*, ∆*Cgmps1* and the complemented strains in picture B and C was indicated. (**B**) Lesions developed on the unwounded detached leaves of poplar inoculated by the wild type SMCG1#C, ∆*Cgmck1*, ∆*Cgmkk1*, ∆*Cgmps1* and the complemented strain ∆*Cgmck1*/*MCK1*. Stars indicate inoculation sites. Photographs were taken five days after inoculation. Scale bars = 10 mm. (**C**) Trypan blue staining of lesions in picture B. Scale bars = 10 mm. (**D**) Invasive hyphae of the wild type SMCG1#C, ∆*Cgmck1*, ∆*Cgmkk1*, ∆*Cgmps1* and the complemented strain ∆*Cgmck1*/*MCK1* developed on the onion epidermic cells. Appressoria, conidia and invasive hyphae, swollen cells were indicated by a, c, ih, and sc, respectively. Photographs were taken 12 h after inoculation. Scale bars = 10 μm. (**E**) Invasive hyphae developed in the poplar leaves in vivo stained by trypan blue. Photographs were taken 12 h after inoculation. Scale bars = 10 μm. Arrows indicated invasive hyphae. (**F**) Penetration ability of the wild type SMCG1#C, ∆*Cgmck1*, ∆*Cgmkk1*, ∆*Cgmps1* and the complemented strain ∆*Cgmck1*/*MCK1* tested on cellophane sheets. Colonies grown for 48 h and 72 h on a CM medium plate covered by a cellophane sheet, and the colony in the same plate with cellophane removal and incubated for one additional day, respectively. ddH_2_O: double distilled water.

To further evaluate the effect of the *CgMCK1* on the invasive structure development in *C. gloeosporioides*, the ∆*Cgmck1* mutant was inoculated on the intact leaves of poplar. The results showed that the ∆*Cgmck1* mutant was no pathogenicity to the plant host (Figure 6A,B). The result of trypan blue staining also showed there were no lesions when leaves were inoculated by the ∆*Cgmck1* mutant. By contrast, apparent necrotic lesions were observed when the leaves were inoculated by the wild type and the complemented strain (Figure 6A,C). These results indicated that the ∆*Cgmck1* mutant completely lost its infection ability to host cells. The similar results were observed when the leaves were inoculated by the ∆*Cgmkk1* and ∆*Cgmps1* mutants.

In order to illuminate the clues of this nonpathogenic phenotype in the ∆*Cgmck1* mutant, penetration assays were performed on the onion epidermal cells. The results showed that the normal appressoria were formed at the end of germ tubes, and further differentiated into invasive hyphae at 18 hpi in the wild type and the complemented strain. However, the ∆*Cgmck1* mutant formed swollen or hooked cells at the end of germ tubes, which further differentiated into vegetative hyphae-like structures rather than invasive hyphae as showed in vitro (Figure 6D). In addition, a large number of invasive hyphae were developed from appressoria in the poplar leaf cell in the wild type and complemented strain. However, there were no invasive hyphae formed when the leaves were inoculated by the ∆*Cgmck1* mutant (Figure 6E). The Invasive hyphae were not developed in the ∆*Cgmkk1* and ∆*Cgmps1*mutants similar to the ∆*Cgmck1* mutant (Figure 6E).

The mycelial penetration ability of the wild type, the ∆*Cgmck1* mutant, and the complemented strains using cellophane membrane penetration assay were examined. The wild type and the complemented strain penetrated the membrane and formed a colony at 48 hpi (Figure 6F). However, the ∆*Cgmck1* mutant took 72 h to cross the membrane, which was similar to the ∆*Cgmkk1* and ∆*Cgmps1*mutants (Figure 6F). These results indicated that CgMck1 was required for appressorium and invasive hyphal development, and penetration to host plant in *C. gloeosporioides*.

### 3.6. *CgMCK1* Deletion Mutant Is More Sensitive to Antagonistic Bacterium Bacillus velezensis

**Figure 7:**
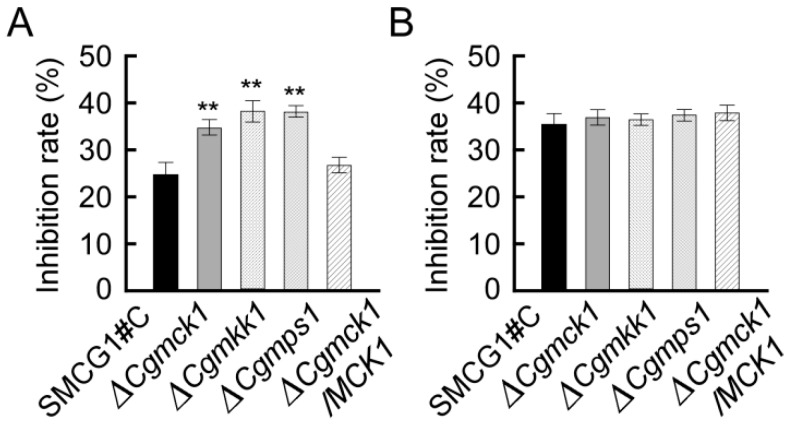
The ∆*Cgmck1* mutant showing higher sensitivity to biocontrol agent *Bacillus velezensis*. (**A**) Antifungal activities of the bacterial biocontrol agent *B. velezensis* against the wild type SMCG1#C, ∆*Cgmck1*, ∆*Cgmkk1*, ∆*Cgmps1* and ∆*Cgmck1*/*MCK1*. (**B**) Antifungal activities of the fungal biocontrol agent *Epicoccum* sp. against the wild type SMCG1#C, ∆*Cgmck1*, ∆*Cgmkk1*, ∆*Cgmps1* and ∆*Cgmck1*/*MCK1*. Error bars represent the SD, and asterisks indicate statistically significant differences (*p* < 0.01).

*Bacillus velezensis* has been shown to be a potential biocontrol agent against a variety of phytopathogenic fungi including *Aspergillus*, *Botrytis*, *Colletotrichum*, *Fusarium*, *Pestalotiopsis*, and *Ralstonia* by secreting cell wall-degrading enzymes to destroy the fungal cell wall [23,25,26,27]. Due to the defects in cell wall integrity in the ∆*Cgmck1* mutant afore-indicated, we hypothesized that *B. velezensis* maybe show a stronger antifungal activity to the ∆*Cgmck1* mutant than that of the wild type and the complemented strain. After four days, *B. velezensis* showed a 38% inhibition of mycelia growth against the ∆*Cgmck1* mutant on the dual culture plates, which was significantly higher than that of the wild type and the complemented strain with 31% and 33% inhibition, respectively (Figure 7A). However, when an antagonistic fungus *Epicoccum* sp. (unpublished) was used as a biocontrol agent to test its inhibition against the wild type, the ∆*Cgmck1* mutant and the complemented strain, there was no significant difference among these strains (Figure 7B). Similar inhibition result was observed in the ∆*Cgmck1* and the ∆*Cgmps1* mutants (Figure 7A,B). These results indicated that the ∆*Cgmck1* mutant was more sensitive to antagonistic bacterial *B. velezensis*, which might be caused by the cell wall integrity defects in the ∆*Cgmck1* mutant*.*

## 4. Discussion

The fungal cell wall serves as an interface between the fungus and the surrounding environment and plays a protective and stereotyped role. MAPK cascades were applied to respond appropriately to environmental changes [6]. The cell wall integrity pathway utilizes one of the MAPK cascades to facilitate the maintenance of the cell wall integrity by mediating cell wall biosynthesis, actin organization, and other events necessary to maintain CWI [28]. In *S. cerevisiae*, CWI MAPK pathway cascade is initiated by cell wall-associated stress sensors Mid2 and Wsc1 [29]. These proteins bind to Rom2, which activates Rho1 and Rho1 activates Pkc1 [30,31], which in turn regulates the MAPK cascade. Pkc1 transmits the signal to Bck1, Mkk1/2 and Slt2 by subsequently phosphorylation [32]. The Slt2 leads to phosphorylation of the transcription factors Rlm1, Swi4 and Swi6, which initiate the expression of cell wall synthase genes [33,34]. The CWI MAPK pathway is conserved among fungi including budding yeast, fission yeast, and filamentous fungi [35].

As a core component of CWI MAPK cascades, the deletion of Bck1 homologue in the human pathogen *Cryptococcus neoformans*, results in marked increases in sensitivity to cell wall stressors SDS and CR [36,37,38]. In *U. maydis*, the deletion of *UmBCK1* leads to sensitivity to cell wall stress stressors [28]. In *M. oryzae*, the deletion of *MoMCK1* results in the serious cell wall integrity defects such as hyphal autolysis [7,8]. Furthermore, *MoMCK1* deletion mutant also showed significantly sensitivity to CR and CFW [7,9]. The phenotypes of its downstream components MoMkk1 and MoMps1 deletion mutants are consistent with the ∆*Momck1* mutant [8]. In this study, the ∆*Cgmck1*, ∆*Cgmkk1*, and ∆*Cgmps1* mutants showed a higher sensitivity when exposed to cell wall inhibitors than that of the wild type and the complemented strain. Apparent hyphal autolysis was observed in the ∆*Cgmck1*, ∆*Cgmkk1*, and ∆*Cgmps1* mutants. Similarly, the loss of these genes also resulted in a faster protoplast release in the ∆*Cgmck1* mutant than that of the wild type.

Previous studies showed that the fungus *Epicoccum* produces active substances such as flavipin, epirodins, epicorazines, and prodiginine to inhibit the growth of plant-pathogenic fungi [39,40,41]. *Epicoccum* sp. strain ENML1 showed similar mycelial growth inhibition between the ∆*Cgmck1* mutant and the wild type on the dual culture plates. *B. velezensis* inhibited the mycelial growth of *Colletotrichum* through extracellular chitinase, protease, and glucanase of *B. velezensis* by damaging the mycelial cell wall [23,42]. In this study, the ∆*Cgmck1* mutant showed significant cell wall integrity defects. The *B. velezensis* also showed a stronger mycelia growth inhibition against the ∆*Cgmck1* mutant than that of the wild type. Combining these results, the data suggested the higher sensitivity of the ∆*Cgmck1* mutant to *B. velezensis* maybe resulted from the defects in the cell wall integrity in the ∆*Cgmck1* mutant.

Like most fungal pathogens, the asexual reproduction plays a key role in the disease cycle of *C. gloeosporioides.* Previous studies showed that sporulation was significantly reduced or even ceased in the ∆*Momck1* and the ∆*Momps1* (MGV1) mutants in *M. oryzae* [7,9]. The deletion of the *MoMKK1* completely abolished the ability to produce conidia [8]. In *Fusarium verticillioides*, FvBck1 was involved in vegetative growth, and conidia production [43]. However, the ability of sporulation in the ∆Fomck1 mutants was similar to the wild type strain [10]. These data indicate that Mck1 may share different regulatory models in the asexual development among plant pathogenic fungi. Here, we found that the deletion mutant of *CgMCK1* significantly reduced the ability to produce conidia and to develop fruiting body on V8 agar plate. Additionally, the vegetative growth was significantly decreased in the ∆*Cgmck1*, ∆*CgMkk1,* and ∆*CgMps1* mutants. These results indicate that CgMck1 and its downstream kinases in the MAPK signaling pathway may be involved in regulating the vegetative growth and sporulation in *C. gloeosporioides*.

In fungal plant pathogens, such as *M. oryzae* and *C. gloeosporioides*, penetration depends on melanized appressoria that drives the penetration peg through leaf cuticles and cell walls. Subsequently, appressoria differentiate biotrophic infection vesicles and primary invasive hyphae. Following the biotrophic stage, necrotrophic secondary hyphae secrete degradation enzymes and kill the host cells [44,45]. In *M. oryzae*, gene deletion mutants of ZNF1 still produce massive conidia. However, these mutants are unable to develop appressoria, and are nonpathogenic [46]. Previous studies showed that appressorium development is regulated by many signal transduction pathways, including the MAPK pathway [47,48,49]. Apposition of melanin in the appressorial cell wall results in a melanin layer, which provides protection against cell-wall-degrading enzymes (CWDE) and helps prevent access of cell-wall-modifying enzymes to structurally relevant cell-wall polymers. The defect in melanin leads to significantly reduced cell-wall stability and, hence, nonpathogenicity [50]. In *C. graminicola*, the deletion of *CgPKS1* results in nonmelanized appressoria and lost the ability to penetrate intact maize and onion epidermis cells [50]. The infection of *C. gloeosporioides* relies on the germination of conidia to produce the germ tube and the tip of a germ tube expands to form an appressorium [16]. In this study, the *CgMCK1* deletion mutant failed to develop an appressorium, although they were able to initiate the swelling of hyphal tips in response to surface hydrophobicity, and the melanin was impaired in these swollen cells. In the wild type, the melanized appressoria drove the penetration peg through leaf cuticles and cell walls, and developed infectious hyphae. However, the ∆*Cgmck1* mutant produced swollen cells at the tip of germ tubes rather than a melanized appressorium. These swollen cells differentiated vegetative hyphae-like structures rather than penetration peg and infections hyphae. These results suggested that *C. gloeosporioides* Mck1 played an essential role in regulating appressorium-mediated plant infection. Previous studies showed that the loss of *MoMCK1*, *MoMKK1* and *MoMPS1* did not result in fully loss of pathogenicity in *M. oryzae* [8]. However, in this study, the deletion of *CgMCK1*, *CgMKK1*, and *CgMPS1* caused to the loss of pathogenicity, which was not consistent with the results in *M. oryzae*. These data indicated that there is a possibility that Mck1 shares a different mechanism to regulate the pathogenicity to plant host among different plant pathogenic fungi.

In summary, CgMck1 plays important roles in regulating the vegetative growth, cell wall integrity, asexual development, appressorium development, and pathogenicity in *C. gloeosporioides*.

## Figures and Tables

**Table 1:** Inhibition rate of mycelia growth of the targeted gene mutants exposed to the cell wall-perturbing agents and osmotic stresses (%).

Strain	0.005% SDS	50 μg/mL CFW	100 μg/mL CFW	200 μg/mL CR	600 μg/mL CR	0.7M NaCl	1M Sorbitol
SMCG 1#C	47.9 ± 0.36 ^B^	12.0 ± 0.8 ^C^	16.9 ± 1.6 ^D^	32.3 ± 2.0 ^C^	49.8 ± 1.0 ^D^	38.1 ± 0.5 ^B^	22.2 ± 1.3 ^B^
∆*Cgmck1*	54.3 ± 2.01 ^A^	20.7 ± 0.6 ^B^	27.9 ± 0.8 ^C^	53.1 ± 0.9 ^A^	53.8 ± 0.2 ^C^	35.4 ± 0.6 ^C^	30.1 ± 1.0 ^A^
∆*Cgmkk1*	55.6 ± 1.04 ^A^	21.8 ± 2.0 ^B^	31.7 ± 0.4 ^B^	42.9 ± 1.0 ^B^	61.1 ± 1.8 ^A^	35.8 ± 0.6 ^C^	26.9 ± 1.0 ^A^
∆*Cgmps1*	51.8 ± 0.22 ^A^	24.5 ± 1.4 ^A^	37.0 ± 3.1 ^A^	42.8 ± 1.0 ^B^	56.5 ± 1.0 ^B^	39.8 ± 0.3 ^A^	27.4 ± 1.0 ^A^
∆*Cgmck1*/*CgMCK1*	49.0 ± 0.38 ^B^	12.3 ± 0.4 ^C^	16.3 ± 0.4 ^D^	32.8 ± 1.0 ^C^	50.5 ± 0.2 ^D^	38.3 ± 0.5 ^B^	24.1 ± 0.6 ^B^
∆*Cgmkk1*/*CgMKK1*	48.1 ± 2.83 ^B^	12.7 ± 1.5 ^C^	17.1 ± 0.6 ^D^	33.1 ± 0.9 ^C^	51.2 ± 3.1 ^D^	36.6 ± 0.4 ^B^	24.8 ± 1.1 ^B^
∆*Cgmps1*/*CgMPS1*	49.5 ± 0.28 ^B^	13.9 ± 0.4 ^C^	19.5 ± 0.4 ^D^	33.3 ± 1.1 ^C^	51.1 ± 0.9 ^D^	37.7 ± 0.3 ^B^	23.2 ± 1.3 ^B^

±SD was calculated from three repeated replicates. Different capital letters indicate significant difference among different strains at *p* < 0.01. SDS: Sodium dodecylsulfate; CFW: Calcofluor white; CR: Congo red.

## Supplementary Materials

The following are available online at http://www.mdpi.com/2073-4425/9/11/543/s1, Table S1: Primers used in this study.
