# Erratum: The MAPKKK CgMck1 Is Required for Cell Wall Integrity, Appressorium Development, and Pathogenicity in *Colletotrichum gloeosporioides* (*Genes* 2018, 9, 543)

**DOI:** 10.3390/genes10100819

**Published:** 2019-10-17

**Authors:** Yu-Lan Fang, Li-Ming Xia, Ping Wang, Li-Hua Zhu, Jian-Ren Ye, Lin Huang

**Affiliations:** Co-Innovation Center for Sustainable Forestry in Southern China, Nanjing Forestry University, Nanjing 210037, Jiangsu, China; fangyulan19921028@gmail.com (Y.-L.F.); summerxlm@163.com (L.-M.X.); pingwangnjfu@gmail.com (P.W.); lhzhu@njfu.edu.cn (L.-H.Z.); jrye@njfu.edu.cn (J.-R.Y.)

The authors wish to make the following correction to this paper [1]. Subfigures i and v were the same in the 12 h panels in Figure 4C.


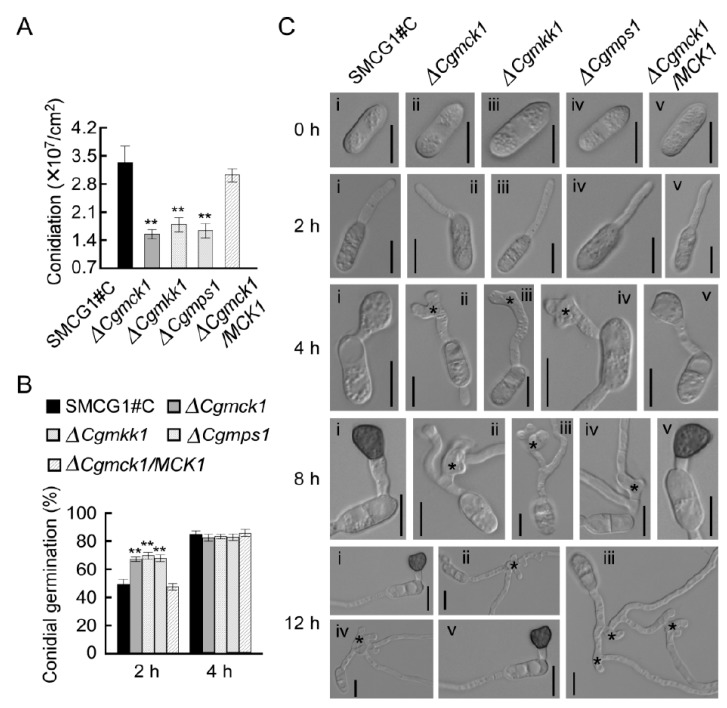


Subfigure v in the 12 h panel in Figure 4C has been correctly replaced in the figure below:


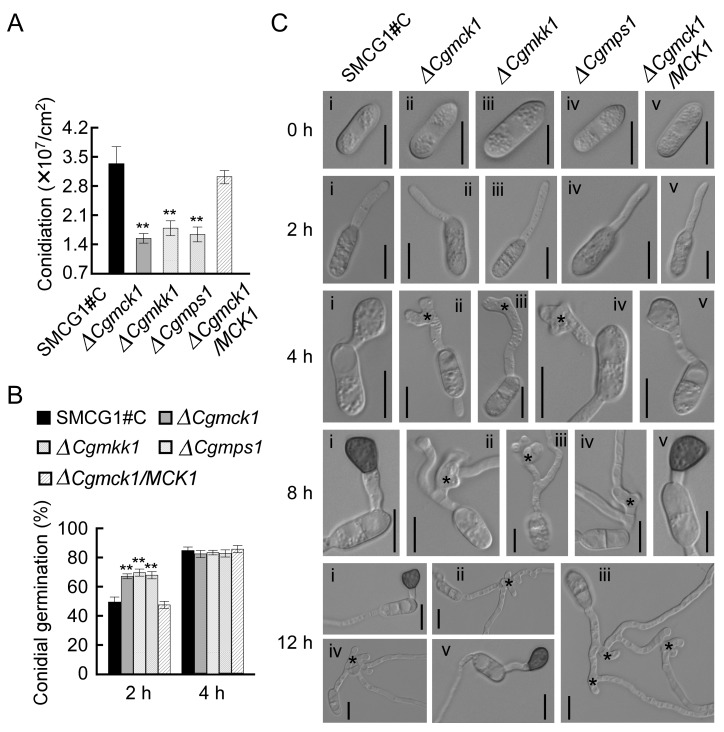


The authors would like to apologize for any inconvenience caused to readers. This change does not affect the scientific conclusions of the article. The manuscript will be updated, and the original will remain online on the article’s webpage, with a reference to this Erratum notice.

## References

[1] Fang Y.-L., Xia L.-M., Wang P., Zhu L.-H., Ye J.-R., Huang L. (2018). The MAPKKK CgMck1 is required for cell wall integrity, appressorium development, and pathogenicity in *Colletotrichum gloeosporioides*. Genes.

