# Supplementary material for: The MAPKKK CgMck1 Is Required for Cell Wall Integrity, Appressorium Development, and Pathogenicity in Colletotrichum gloeosporioides
Source: Genes (Basel). 2018 Nov 8;9(11):543. doi: 10.3390/genes9110543 (PMC6267176; doi:10.3390/genes9110543)
Supplement: Supplementary file 1 [file genes-09-00543-s001.pdf]

Table S1. Primers used in this study

Primer name	Sequence (5'-3')
CgMCK1_F1	ACTTGAGGCTGGACTGTATG
CgMCK1_R1	TTGACCTCCACTAGCTCCAGCCAAGCCGGTACAGTGCCAATGTCGT
CgMCK1_F2	GAATAGAGTAGATGCCGACCGCGGGTTCGCGAGGGGAACTCAGTA
CgMCK1_R2	CATACATGCCATCAAGCAATC
CgMCK1_F3	ACCTTAGTCTCCCGTCTGC
CgMCK1_R3	ACAAAATCACCCGCATCA
CgMCK1_comF	CAGAGTGAGTGCGGATTCAG
CgMCK1_comR	GGTATCTCGCACCTTGACATG
CgMCK1_InnerF	GCATGTCTGCGACCTACATAC
CgMCK1_InnerR	CTGTTGAGGGTAGGCATAGT
CgMKK1_F1	CAGGTACAGCCCAAGTGAC
CgMKK1_R1	TTGACCTCCACTAGCTCCAGCCAAGCCAAGGCGATACGATAAGCAG
CgMKK1_F2	GAATAGAGTAGATGCCGACCGCGGGTTAGCGACCAAGTCGGCAATA
CgMKK1_R2	AGCAGGCGAACAAGAAGG
CgMKK1_F3	ATGTAAGGCGCAATTAAGG
CgMKK1_R3	GAGACGATGGAGGATGCTG
CgMKK1_comF	CGATTTCGGTCCATGCTTCAAG
CgMKK1_comR	TGTACCTCCACGCCTACCAG
CgMKK1_InnerF	TACCGACCCTTCACCTTGC
CgMKK1_InnerR	CGTCGGATGTGATGGTGTATG
CgMPS1_F1	CGGTGGGATAAGGTGGCT
CgMPS1_R1	TTGACCTCCACTAGCTCCAGCCAAGCCTTTGGATCGGTTGGTGAA
CgMPS1_F2	GAATAGAGTAGATGCCGACCGCGGGTTACGAAAAGATTCTGTGCG
CgMPS1_R2	GGTTGACCTACCGTGCCT
CgMPS1_F3	GATAAGGTGGCTCCTTCG
CgMPS1_R3	GCACTCACGCCATATTCAAC
CgMPS1_comF	CTGAATGCGAGGCGACTC
CgMPS1_comR	CTTGGACGCGTGCTCATG
CgMPS1_InnerF	ATACACCGTCACCAAGGAGC
CgMPS1_InnerR	GGGAAAGGCTTCTTAGGC
YG_F	GATGTAGGAGGGCGTGGATATGTCCT
HY_R	GTATTGACCGATTCCTTGCGGTCCGAA
HYG_F	GGCTTGGCTGGAGCTAGTGGAGGTCAA
HYG_R	AACCCGCGGTCGGCATCTACTCTATTC
